# Medical Society of the County of Albany

**Published:** 1876-01

**Authors:** 


ART. II.—*Medical Society of the County of Albany.* Semi-monthly Meeting, Dec. 15th, 1875.

The President, Dr. HENRY MARCH, in the Chair.

Dr. JAMES S. BAILEY then reported the following case of eclampsia occurring during the eighth month of pregnancy. He said: Mary K., æt. 42, Irish, Sept. 20th, 1875, came to engage me to attend her in confinement. She had been twice married. Eight years previous had had one child by her first husband, the labor of which was normal. Her skin was pale and transparent, which led me to suspect albuminuria. Upon examination, I found her feet and limbs swollen slightly.

The next day her urine was found to be highly albuminous. About dark the same day she had an attack of acute pain in the epigastrium. An anodyne procured relief and sleep. Towards day she was seized with eclampsia. I saw her in the second spasm, which was very severe; when this had passed off, she was rational, but could not see.

During the next twelve hours she had four spasms, which left her unconscious, and she was muttering in delirium. She had passed but a small quantity of bloody urine. I gave her bromide of potassium, ten grains every two hours, which relieved the spasms. The administration of ten grains of calomel operated freely on her bowels.

The next day she was rational, but could not see. The pulse was frequent and feeble, with coolness of the extremities. There had been no motion of the child since the first spasm.

September 24. Is rational. Her condition is much improved, and she is inclined to joke, but cannot see. The right leg and arm are swollen considerably, and she cannot use them. The circulation is better. The urine is scant and high-colored. I was enabled now, for the first time, to procure urine for examination, which was solid with albumen. The bromide was continued, and she drank freely of parsley-root tea.

September 25. She cannot distinguish a person standing at the foot of the bed; has passed one-half gallon of urine during the past twenty-four hours.

September 26. Passed one and one-third pints of urine; is improving.

September 27. To-day she realizes the seriousness of her condition, and asks many questions in reference to the probability of passing safely through her confinement without spasms.

September 28. The swelling has left her face and limbs; her vision has improved, and she can now turn over in bed without assistance, and has passed during the day one gallon of urine; treatment continued.

September 29. Says that she is well.

September 30. To-day she sat up for the first time.

October 1. Labor commenced at 9 A. M.; by 12 M. the os uteri was fully dilated; the membranes ruptured, disclosing a foot presentation. The birth was soon accomplished, excepting the head, which was large, and as the fœtus was much decomposed, I feared to make much traction for fear of severing the head from the trunk. The forceps were applied, and the delivery completed by 12.30 o'clock.

There were no more spasms, and she continued to improve rapidly.

Upon relating the case to professional friends, I was advised to immediately induce labor, but as she seemingly was progressing favorably, I determined to wait for labor to set in naturally, and if eclampsia appeared, to deliver at once.

The happy termination of the case, I think, proved my judgment correct.

During the past quarter of a century much light has been thrown upon albuminuria accompanying the lying-in state.

The recent progress of pathology has directed the attention of physicians to the kidneys instead of the brain. Here abundant cause is found for the cerebral symptoms. The great advantage gained from the progress in our investigations has been by way of prophylaxis. Though the mortality continues large, it is a satisfaction to know that death from this cause is unavoidable. It has been my custom to attentively examine the urine of pregnant women engaging my services for accouchment, especially when there were evidences of albuminuria.

During the last five hundred accouchments in which the urine was examined, I find twenty-eight cases in which albumen was found in varying traces. In six of these the albumen depended on the admixture of pus. Deducting these, we have a ratio of one in nearly twenty-two cases. This complication existed in large proportion among primiparæ. For such investigations the urine should always be drawn with a catheter, to insure freedom from admixture from vaginal secretions. The physician learns at last, after patient investigation, and long experience that the urine of pregnancy may present, microscopically, all the varieties and numbers of casts which are recognized in the different stages of Bright's disease, yet after the successful lying-in period has passed these threatening symptoms may entirely disappear, and the patient recover her wonted health.

The difficulty in rendering a correct prognosis is therefore apparent, and here the hopes and fortunes of the younger physician may be wrecked by prognosticating a positive fatal termination, when the experience of riper years would change his opinion, if not make him more cautious in expressing it.

Such cases teach us a valuable lesson; for while we have escaped one difficulty, we are warned of another which may be not far distant. Such serious, renal encroachments are apt to be followed in future pregnancies; therefore, when such is the case, watchfulness and frequent examinations of the urine is demanded. According to my experience, primiparæ are more liable than multiparæ to albuminuric-eclampsia, but the graver consequences are most commonly settled on the latter. Mercurial purges are very beneficial in some cases. I use calomel in full doses, followed with bark and iron, especially when the head symptoms are absent and hydræmia well marked.

The salines for prophylactic treatment have with me answered an excellent purpose, such as Rochelle salts, citrate of magnesia, Seidlitz powders, or even the mineral waters of Saratoga.

The condition of the skin should always be taken into consideration. When there is a hot, dry surface, a mustard pedeluvia given in bed operates speedily in obtaining diaphoresis; then diaphoretics and diuretics come in admirably, and it is always best to select those that are least stimulating. When there is sleeplessness and excitability of the nervous sytem, I give bromide of potassium. The tea of parsley root taken freely will, so to speak, wash the kidneys thoroughly, which in my opinion is absolutely necessary to save the patient from the toxical effect.

The points of interest in this case are as follows: The degree of health experienced by her, even up to the time of spasms, she not having suspected any trouble, she said that her health was perfect—that she never felt better. That the poison was sufficient to destroy the child, and yet the mother perfectly recovered.

The variation in the quantity of urine secreted every twenty-four hours is also surprising, varying in quantity from two ounces to one gallon by actual measurement; also in the quantity of albumin contained in the urine each day.

Such cases are rarely met with, especially when recovery takes place. A tabulated statement of the urine is here presented, showing its condition during the most trying period of her sickness:

| Dates. | Reaction. | Sp. Grav. | Amt. of Albumen. | Appearances. | Quantity passed per day. | Microscopic Appearance. |
|---|---|---|---|---|---|---|
| Sept. 21 | Acid | 1015 | 1-5 | Normal | | E R. Epithelium, no casts. |
| " 22 | Alkaline | 1030 | Solid | Dark Red | 2 oz. | Graular and Hyaline casts, E. R. Epithelium, Blood Globules. |
| " 24 | Acid | 1015 | 1-2 | " | 1-2 pt. | Hyaline and Granular casts, Epithelium. |
| " 25 | " | 1013 | 1-3 | Normal | 1-2 gal. | Uric Acid Crystals, Epithelium and Granular casts. |
| " 26 | " | 1018 | 1-3 | " | 1 1-3 pts. | Degenerated Renal Epithelium, Uric Acid, Granular casts. |
| " 27 | " | " | 1-4 | " | 1-2 gal. | Granular Sates, Epithelium, Uric Acid. |
| " 28 | " | " | 1-8 | " | 1 gal. | Granular and Hyaline casts, Renal Epithelium. |
| " 29 | " | " | 1-6 | " | 3 qts. | Hyaline and Granular Casts, Epithelium. |
| " 30 | " | 1022 | 3-4 | " | 3 pts. | A few Hyaline casts, |
| Oct. 1 | " | 1010 | 1-4 | Dark Amber | --------- | Blood Globules abundant, Hyaline and Granular casts. |
| " 2 | " | " | 1-3 | Normal | --------- | Blood Globules and Hyaline casts. |
| " 3 | " | " | 1-6 | Redish Deposit | --------- | Blood. |
| " 8 | " | 1025 | 1-3 | Normal | --------- | No casts, E. R. Epithelium, Blood Globules. |
| " 18 | " | 1023 | Faint Trace | Amber | --------- | Precipitate abundant, E. R. Epithelium, one fatty cast found with Epithelium attached. |
| " 21 | " | 1030 | " | " | --------- | E. R. Epithelium abundant. |
| " 25 | " | 1012 | Free | " | --------- | No casts, but little Epithelium. |

Dr. HANNAN enquired what proof the Doctor had that the albumen produced the eclampsia. Might it not be due to an excess of urea? Did not believe that albumen could produce eclampsia, and asked how often the Doctor or any gentleman in the room had seen albuminuria in the numerous cases of Bright's disease which had come under their care.

Dr. WM. H. BAILEY asked Dr. Hannan if cold or congestion of the kidney's was not liable to bring on albuminuria.

Dr. HANNAN replied that the kidneys had been removed, and still albumen was found.

Dr. MUNSON enquired how long the loss of sight had continued, and how it came on.

Dr. JAMES S. BAILEY replied it came on suddenly after the first spasm and lasted three days, but now the patient has perfect vision.

Dr. DAVIS enquired how far she was advanced in pregnancy.

Dr. JAMES S. BAILEY said between seven and eight months, at least so the patient said when she applied at his office.

Dr. DAVIS said there are so many interesting questions in the study of eclampsia and no class of diseases are of more importance to the physician. So far as the pathology goes, he was not very well informed, but was taught to consider them as no more dangerous than like convulsions occurring in other diseases.

The treatment should be directed chiefly to the brain, to save the condition of the brain. The Doctor was of the opinion that the convulsion differed in character in resembling respectively hysteria epilepsy and apoplexy. The labor would take care of itself. Our attention should be directed to the convulsions. If the case resembled hysteria treat accordingly. If epileptiform do likewise. If it partook of the nature of apoplexy, bleed. In such a case he thought bleeding the sheet anchor. Such was his treatment and he had never lost a case in his own practice, but had seen death occur from this cause in the practice of others. When the symptoms are relieved, then, if you can, deliver.

Dr. WM. H. BAILEY inquired how Dr. Davis distinguished between the cases?

Dr. DAVIS replied as in many cases of convulsions. The movements are characteristic, somewhat like epilepsy, and epilepsy may resemble apoplexy, but the history of the case the movements of the patient, the character of the convulsions will generally show the nature of the case.

Dr. HANNAN said Dr. Davis referred to Venesection, he would like to know on what principle does a man bleed? If it was to lessen the calibre of the blood vessels.

Dr. C. D. MOSCHER said it is well to make sure of the diagnosis of these cases. The great similarity of the symptoms makes one doubt as to the character of the case. The Doctor was inclined to differ from Dr. Bailey as to the diagnosis of the case under discussion. He thought it rather a case of hysteria than of eclampsia and that any slight shock falling on the system in the condition the patient was it would cause an attack.

The Doctor thought the non examination by the opthalmoscope was a loss to the diagnosis, and that therefore the loss of vision was a deceptive symptom. The treatment though successful he did not think was the kind to be followed. Bleeding would have been in this case a serious mistake, but judging from the treatment and the result, does not think it a case of eclampsia.

Dr. CURTIS said in relation to the question of bleeding which had been spoken of, he had had lately a chance to see the good effects of such treatment. He saw a case which had been treated by Dr. Boyce when convulsions came on suddenly, six in all, taking place rapidly. The patient had bitten her tongue, and was in the usual condition of such cases. Dr. Boyce bled the patient through a large opening and drew sufficient blood to fill an ordinary tin wash basin. The effect was almost immediate, and when he saw the patient again she could hardly be said to be sick.

The loss of vision he had seen in one case before; it lasted for some days, but passed off without trouble. He hoped some one would say something about the cause of this symptom. In the case of Dr. Bailey he thought the trouble came from the kidneys. Casts showed themselves in the urine which showed the kidneys to be in a state of acute Bright's disease. Roberts states that two-thirds of the cases of acute Bright's disease recover. In this case there were large amounts of granular and hyaline casts and some blood. The Doctor did not think the cause of the convulsions was the albumen but was undoubtedly the circulation in the blood of some substance which the kidneys should have cast off.

Dr. HANNAN said he had bled a patient some thirty ounces and it made no impression. He doubted also whether urae produces spasms. If anything thinks it is the carbonate of ammonia in the blood. Pathologists, physiologists and chemists have found carbonate of ammonia in the circulation instead of urea.

Dr. STONEHOUSE referred to a case of Bright's disease reported to the Society by Dr. Ullman where there was eclampsia, and also loss of vision.

Dr. STONEHOUSE presented the following case of *Syphilitic Meningitis:*

The Docter said: J. G. M., æt. 26, applied to a physician in New York City for treatment of an excavated syphilitic ulcer of the throat, but was with difficulty convinced that it was necessary to continue treatment after the cure of the throat affection, and two weeks after that date he broke off attendance before he was dismissed. In three months he returned suffering from syphilitic iritis of both eyes but no eruption.

Before his complete recovery he again returned to his work and discontinued treatment. His physician saw nothing of him for several months when he was summoned to attend a gentleman at a hotel near his office, who had been attacked with hemiplegia of the right side while he was returning to his home in New Jersey, and found his former patient. On the next day I saw him for the first time with his physician, and from his partner who was with him on the train, we ascertained the following history of the three days previous:

He had complained of dull, persistent pain over the anterior portion of the cranium and extending backward midway to the occiput. His face had been considerably flushed, and the conjunctiva very much injected. The head had been quite hot. He had vomited once but a few hours previous to the paralytic attack. He was exceedingly restless, and had not slept any during the four days previous to this attack. The heat of the skin, and the flushing of the face being greatly increased during the night. On the morning of the attack he had talked somewhat irrationally, and his partner was fearful to undertake the journey with him lest he should attempt self destruction. He took the train at 9 A. M., arriving in New York at 12:15 P. M., having been attacked with hemiplegia fifteen minutes previous to his arrival. At the time of our visit he had rallied from the attack, but the headache, suffusion of countenance and high temperature of the surface were all increased. Active delirum had set in. Curiously he seemed to suffer from no photophobia or morbidly acute hearing, although the character of the two most prominent delusions would appear to indicate their presence. He complained almost continually that his attendants were burning out his eyes with red hot irons, and trying to deafen him by the marvelously loud noises they were making; although, perhaps the foundation for the first delusion was the admission of a little extra light through the door, or for the other delusion the falling of a spoon upon the floor, or the step of a very careful and experienced nurse. His pulse was quick and thread-like; he had great thirst, loss of appetite and obstinate constipation, his bowels not having moved for three days. He was extremely sensitive to the least impression upon his skin, complaining sometimes of the bed clothes. There were twitchings of the facial muscles and irregular movements of the eyeballs.

By my advice mercurial inunction was made in both axillæ, and upon the shaven scalp. An active purgative was administered and the following ℞ made:

Potassi Iodidi, ℥i.

Aquæ ℥iv. m.

Sig. Cap. ʒi. ter in die.

Cold applications to the head to be constantly applied. In three days I saw the patient again and found him much better. The pulse was slower and fuller, the delirum had subsided, the conjunctiva less injected, and the pain relieved. Paralysis much the same. The mercurial was discontinued and a tonic administered with the iodide. In two days more he was entirely sane, the paralysis disappearing and other symptoms either entirely stopped or much diminished in severity. At the end of a week he called upon me with no paralysis or meningeal symptoms, but suffering from extreme weakness which, however, I learned soon passed off, and the patient had been entirely free from any constitutional manifestations.

Dr. HANNAN enquired what was the proof that these symptoms was caused by syphilis.

Dr. STONEHOUSE answered the only proof is the previous history and that the patient was a very careful man, guarding himself against taking cold. The course of the disease, but mainly the result of treatment.

Dr. VAN DERVEER asked if the patient gave any history of a primary sore?

Dr. STONEHOUSE replied he did to his physician. Had it before the sore throat, but the Doctor said he did not know how long he was under treatment for the sore throat, nor whether the treatment was mercurial.

Dr. MOSCHER asked how long it was since the first attack?

Dr. STONEHOUSE did not know.

Dr. MUNSON asked if the iritis lasted two months?

Dr. STONEHOUSE re-read that portion of the case relating to the history.

Dr. VAN DERVEER said he regretted the history was not brought up to a later date. In his experience he had found these cases required a long course of treatment. The mercurials must be continued for a long time.

Dr. STONEHOUSE said he did not know how long the treatment had been continued but the patient was now well.

Dr. VAN DERVEER enquired how many cases of hemiplegia due to syphilis.

Dr. STONEHOUSE said he did not know; had seen no statement on the subject.

Dr. DAVIS said Dr. Bumstead spoke of a case similar to the one related by Dr. Stonehouse in which there were several relapses. In his own later experience of syphilis he did not rely only on the Iodide of Potassium, but mainly on the use of mercurials.

Dr. VAN DERVEER asked Dr. Davis how soon would he use mercury with a primary sore.

Dr. DAVIS said he would not use it at all with the primary sore, but would wait until secondary symptoms showed themselves.

Dr. MOSCHER said that he had had a patient where similar symptoms showed themselves caused by syphilis. Large doses of the

Iodide of Potassium changing sometimes to the Iodide of Sodium relieved the patient. Sometimes the treatment was stopped altogether, He knew nothing of the history of the patient previous to his treatment of the case about two years since. The patient became insane early and only recovered her mind on taking large and continued doses of the iodide. Judging from that case he thought it necessary to continue the treatment for a long time.

Dr. MUNSON said he considered it important how the mercurial treatment was applied. He thought the best method is by way of inunction and hyperdermic injections. In this way a patient can be brought under the influence of the drug in three or four days.

Dr. STONEHOUSE said that he had seen several cases of insanity from syphilis which recovered under treatment and left the asylum. In his experience the iodide with the use of mercurials by inunction was the best method of treatment.

Dr. WM. H. BAILEY enquired of Dr. Van Derver whether he would use constitutional treatment during the primary lesion.

Dr. VAN DERVER said that he would not.

Dr. HALE said he would like to ask concerning the large doses of the Iodide of Potassium. If ʒi. three or four times a day would be allowable.

Dr. MOSCHER said that he had read of such doses being used, but had had no experience in that way. He thought sufficient doses should be used to procure the effect sought.

Dr. DAVIS said he did not know why it was, but he had been unsuccessful in using large doses of the Iodide. When he even got as high as ten to fifteen grains he had trouble from the stomach.

The President refered to the peculiar ptyalism which came on after the use of large doses of Iodide.

Dr. STONEHOUSE said the largest doses he had seen given was in those cases when it was administered for the cranial pains.

Dr. HALE asked if ʒi. as a dose did not produce gastric trouble, would constitutional trouble be likely to follow.

Dr. DAVIS said he had no experience to guide him, but would refer the Doctor to the literature on the subject. He had however had a case where ptyalism was the result of continued use of the drug, and it was so bad that the patient would wet the pillow at night, rendering it necessary for him to discontinue treatment.

Dr. WEIDMAN suggested that mercury might have been used before by the patient, and the ptyalism be due to that.

The Society then adjourned.

